# Ghrelin is an Osteoblast Mitogen and Increases Osteoclastic Bone Resorption In Vitro

**DOI:** 10.1155/2011/605193

**Published:** 2011-09-08

**Authors:** Jessica L. Costa, Dorit Naot, Jian-Ming Lin, Maureen Watson, Karen E. Callon, Ian R. Reid, Andrew B. Grey, Jillian Cornish

**Affiliations:** Department of Medicine, University of Auckland, Private Bag 92019, Auckland 1142, New Zealand

## Abstract

Ghrelin is released in response to fasting, such that circulating levels are highest immediately prior to meals. Bone turnover is acutely responsive to the fed state, with increased bone resorption during fasting and suppression during feeding. The current study investigated the hypothesis that ghrelin regulates the activity of bone cells. Ghrelin increased the bone-resorbing activity of rat osteoclasts, but did not alter osteoclast differentiation in a murine bone marrow assay nor bone resorption in ex vivo calvarial cultures. Ghrelin showed mitogenic activity in osteoblasts, with a strong effect in human cells and a weaker effect in rat osteoblasts. The expression of the human ghrelin receptor, GHSR, varied among individuals and was detectable in 25–30% of bone marrow and osteoblast samples. However, the rodent *Ghsr* expression was undetectable in bone cells and cell lines from rat and mouse. These data suggest that elevated levels of ghrelin may contribute to the higher levels of bone turnover that occurs in the fasted state.

## 1. Introduction

Bone is constantly remodeled through the actions of bone-forming osteoblasts and bone-resorbing osteoclasts. Bone turnover is acutely responsive to food intake, with bone resorption increased during fasting and suppressed during feeding [1, 2]. Numerous factors, such as nutritional content or size of meals, may play a role in this relationship and there is evidence that some gut hormones, such as glucagon-like peptide-2 [3, 4], also affect the normal postprandial reduction of bone resorption.

Ghrelin is a 28-amino-acid peptide hormone primarily synthesized by the stomach and released in response to fasting, such that circulating levels, which range from 100–120 pmol/L in rats [5] or 90–240 pmol/L in humans [6], are highest immediately prior to meals and fall upon feeding [7, 8]. Two forms of ghrelin are found in the circulation: n-octanoyl ghrelin, which is acylated with an n-octanoyl group on serine residue 3, and des-acyl ghrelin [5]. The n-octanoylated form of ghrelin binds the GH secretagogue receptor (GHSR1a), increases food intake [9–11], and induces release of growth hormone from the pituitary via the hypothalamus [5, 12] as well as by directly acting on the pituitary [5]. The major form of circulating ghrelin is des-acyl ghrelin, which does not signal through GHSR1a and was considered to be inactive, although recent studies suggest that des-acyl ghrelin has some biological activity [13, 14]. The ghrelin receptor, GHSR, is a seven transmembrane-domain G-protein coupled receptor [15]. Two transcripts are produced from the human GHSR gene by alternative splicing: GHSR1a encodes the functional receptor, and GHSR1b, which is truncated after transmembrane domain 5, appears to be unable to stimulate intracellular signaling pathways [15]. 

The activity of ghrelin to stimulate feeding produced a great interest in this peptide as a potential drug target. Receptor antagonists or inverse agonists have been developed for potential treatments of obesity by the induction of satiety [16, 17] and ghrelin and receptor agonists have been considered for treatment of cancer-related cachexia [18, 19]. In addition, ghrelin has been investigated for a therapeutic role in cardiovascular disease [20, 21] because of the expression of the ghrelin receptor in vascular smooth muscle cells of the cardiovascular system [22]. 

A number of studies investigated the effects of ghrelin in skeletal tissue. Expression of the ghrelin receptor, GHSR1a, has been demonstrated by reverse transcription-PCR, immunohistochemistry, and western blot analysis in several osteoblasts and osteoblastic cell lines [23, 24] and a study of the expression of GHSR1a in human tissues demonstrated a weak positive signal in bone marrow [25]. In vitro studies showed effects of ghrelin in primary osteoblast-like cells and cell lines, with highly variable responses in proliferation, differentiation, and survival assays [23, 24, 26–28]. Osteoclast response to ghrelin has never been investigated previously. However, as a growth hormone secretagogue, ghrelin is likely to have an indirect effect on osteoclasts, as growth hormone increases formation and function of osteoclasts isolated from mouse or rabbit bone marrow or spleen [29]. 

The aim of the current study was to further investigate the activity of ghrelin in bone cells, and in particular its possible role in the acute stimulation of bone resorption that occurs during fasting. We used assays of both osteoclast formation and mature osteoclast function to investigate ghrelin's role in bone resorption, in addition to testing the effects of ghrelin on osteoblasts, and investigating the expression of the ghrelin receptor in bone cells.

## 2. Methods

### 2.1. Materials

Acylated ghrelin with n-octanoyl at serine residue 3 was purchased from American Peptide Company (Sunnyvale, CA) and this form was used exclusively in all studies. BSA was purchased from ICPbio International, Ltd (Auckland, New Zealand). Gibco brand FBS and media were used (Invitrogen, Auckland, New Zealand). Tritiated thymidine was purchased from NZ Scientific, Ltd (Auckland, New Zealand). Tartrate-resistant acid phosphatase (TRAP) staining kit, 1,25-dihydroxyvitamin D3, osteoprotegerin (OPG), and salmon calcitonin were purchased from Sigma (NSW, Australia).

### 2.2. Cell and Tissue Samples from Human Subjects and from Animals

Animals were purchased from the Vernon Jensen Unit of the University of Auckland. All animal procedures were approved by the Animal Ethics Committee of our institution. Human bone samples were collected from consenting subjects undergoing hip and knee arthroplasty for arthritis. The study had the approval of the local institutional review board.

### 2.3. Primary Osteoblast Proliferation, Differentiation and Apoptosis Assays

Rat osteoblasts were isolated from 20-day fetal rat calvariae, as previously described [30, 31]. For proliferation assays, cells were seeded into 24-well plates in 5% FBS/MEM/5 *μ*g/mL ascorbic-acid-2-phosphate (AA2P) for 24 h. Cells were growth-arrested in 0.1% BSA/MEM/5 *μ*g/mL AA2P for 24 hours. Fresh media and experimental compounds were then added for a further 24 hours. Cells were pulsed with ^3^H-thymidine 6 hours before the end of the experimental incubation. The experiment was terminated and cell counts or thymidine incorporation determined. There were six wells in each group, and each experiment was repeated three or four times. Mineralization and apoptosis assays were carried out on this cell type as previously described [31]. Mineralization was determined by von Kossa stain, and apoptosis by terminal deoxynucleotidyl transferase-mediated deoxyuridine triphosphate nick end labeling (TUNEL). 

Cultures of primary human osteoblasts were prepared using normal human trabecular bone. Osteoblasts were grown from enzyme-treated bone chips, using a modified method of Robey and Termine [32] and treated with experimental compounds 24 or 48 hours prior to end of the incubation. The experiments were carried out three times with similar results.

### 2.4. Bone Marrow Culture

Bone marrow was obtained from the long bones of normal Swiss CD-1 male mice aged 4–6 weeks, as previously described [30, 31]. Briefly, marrow cells were grown in 48-well plates and on days 0, 2, and 5, 1,25-dihydroxyvitamin D3 (10^−8^ M) was added to all wells. On days 2 and 4, cultures were fed with 0.5 mL fresh medium containing test substances. Recombinant human osteoprotegerin (10 ng/mL) was included as a positive control for inhibition of osteoclastogenesis. After 7 days of culture, TRAP-positive multinucleated cells (containing three or more nuclei) were counted in all wells. There were at least eight wells for each group, and the experiment was carried out four times with similar results.

### 2.5. Mature Isolated Osteoclast Culture

Rat osteoclasts were isolated from the long bones of 1-2-day-old rats, as previously described [30, 31] and incubated on bovine bone slices with test substances or vehicle for 24 hours. After incubation, the bone slices were fixed and stained for TRAP. The potent inhibitor of osteoclast activity, salmon calcitonin (10^−10^ M), was used as a positive control. The number of TRAP-positive multinucleated cells containing three or more nuclei on each bone slice was counted, the cells were removed by gentle scrubbing, and then the bone slices were stained for 30 seconds with toluidine blue. After several washes in water, the bone slices were dried and assessed for the pits excavated by the osteoclasts, using reflected light microscopy with metallurgic lenses. The results for each bone slice were expressed as the ratio of the number of pits to the number of osteoclasts. There were 6–8 bone slices in each group and the experiment was carried out more than three times.

### 2.6. Bone Organ Culture

Hemicalvariae labeled in vivo with ^45^Ca were collected as described previously [31] and incubated with experimental substances or vehicle for 48 hours. Calcium release was assessed and used to calculate the percent of ^45^Ca released over total ^45^Ca injected per animal (3 *μ*Ci), and ^3^H-thymidine incorporation was measured as an indicator of cell proliferation. Parathyroid hormone (PTH) (10^−8^ M) was used as a positive control. There were five to seven hemicalvariae in each group, and the experiment was carried out more than three times.

### 2.7. RNA Isolation, Reverse Transcription, and Quantitative PCR 

RNA was collected from flash frozen mouse hypothalamic tissue or cultured cells with Qiagen RNeasy minikits, and genomic DNA was removed using the RNase-free DNase set (Qiagen) according to manufacturer's instructions. RNA samples collected from cell cultures grown from human osteoblast and bone marrow have been previously described [33]. cDNA was synthesized with Superscript III (Invitrogen) and used for multiplex real-time PCR in ABI PRISM 7900HT Sequence Detection Systems (Applied Biosystems). Primers and probe sets were purchased as TaqMan Gene Expression Assays (Applied Biosystems) for mouse *Ghsr*, rat *Ghsr,* and human GHSR. All probes used to detect target genes were labeled with FAM and 18 S rRNA endogenous control probe was VIC labeled. The ΔΔCt method was used to calculate the relative levels of expression [34].

### 2.8. Statistics

Data were analyzed using ANOVA with post hoc Dunnett's tests or Student's *t*-test as indicated for experiments with 2 or more treatments. Student's *t*-test was used where a single treatment was analyzed versus vehicle. A 5% significance level (*P* < 0.05) is used throughout. Data are presented as means ± SEM, unless indicated otherwise.

## 3. Results

### 3.1. The Effects of Ghrelin on Osteoclast Differentiation and Activity

**Figure 1:**
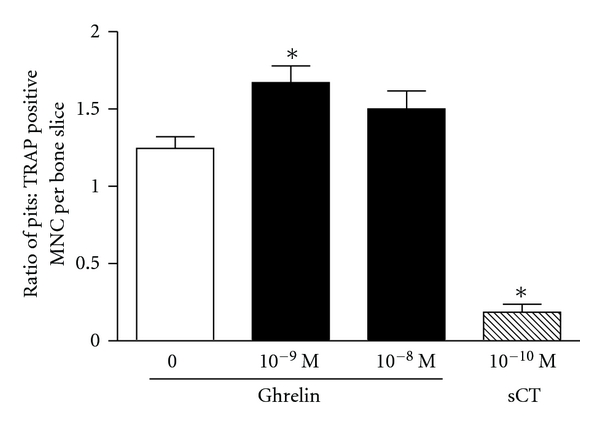
Ghrelin increases bone resorption by mature rat osteoclasts. Osteoclasts were incubated on bovine bone slices for 24 hours, and the number of TRAP-positive multinucleated cells on each bone slice was counted. Cells were then removed, the bone slices were stained with toluidine blue, and the number of excavated pits was determined by reflected light microscopy. The results are expressed as the ratio of the number of pits to the number of osteoclasts on a bone slice, with 6–12 bone slices in each group. MNC: multinucleated cells. sCT: salmon calcitonin (positive control). Data are mean ± SEM; *indicates *P* < 0.05 compared to untreated control.

**Figure 2:**
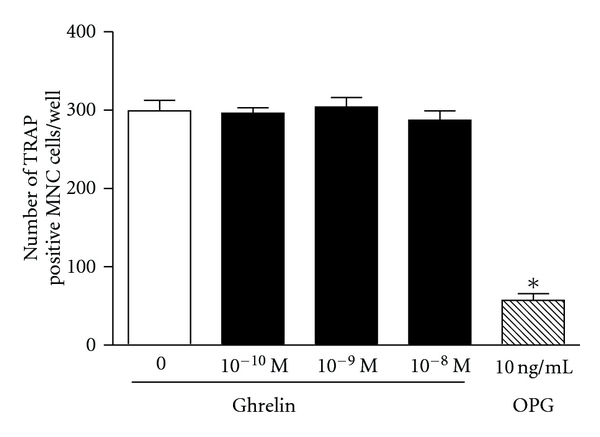
Osteoclast differentiation in mouse bone marrow cultures is not affected by ghrelin. Bone marrow cells were cultured for 7 days with experimental substances or vehicle. Cells were fixed and stained and the number of TRAP positive cells with three nuclei or more was determined in each well. MNC: multinucleated cells, OPG: osteoprotegerin (positive control). Data are mean ± SEM; *indicates *P* < 0.05 compared to untreated control.

**Figure 3:**
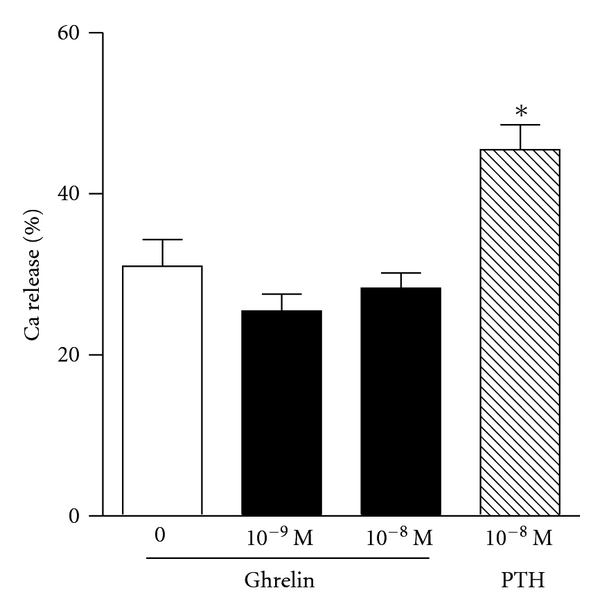
Osteoclast activity in mouse calvarial culture is not affected by ghrelin. Calvariae labeled in vivo with ^45^Ca were collected and incubated ex vivo with experimental substances or vehicle for 48 hours. Values are expressed as percent released ^45^Ca detected over total ^45^Ca injected (3 *μ*Ci). PTH: parathyroid hormone (positive control). Data are mean ± SEM; *indicates *P* < 0.05  compared to untreated control.

We used a number of in vitro experimental systems to study the effects of ghrelin on osteoclast differentiation and activity. Mature osteoclasts were isolated from long bones of 1-2-day-old rats and cultured on slices of bovine bone. Ghrelin at a concentration of 1 nM induced a 30% increase in the number of pits excavated by osteoclasts, whereas at 10 nM there was only a 15% increase which was not significantly different from the control (Figure 1). Osteoclast differentiation was investigated using a murine bone marrow assay, where the mixed population of cells from the bone marrow is cultured in the presence of 1,25-dihydroxyvitamin D3 that promotes osteoclast formation. The addition of ghrelin at concentrations of 0.1–10 nM during the seven-day incubation period had no effect on the number of osteoclasts as compared to control (Figure 2). The activity of ghrelin was also tested in an ex vivo organ culture of mouse calvariae. This experimental system is investigating osteoclast activity rather than development as there is only a minimal number of preosteoclasts in the calvariae. The ability of osteoclasts to resorb bone, as measured by %  ^45^Ca release, was unaffected by ghrelin (Figure 3). Measurement of ^3^H thymidine incorporation in the same cultures showed no difference in cell proliferation in the presence of ghrelin (data not shown). 

### 3.2. The Effects of Ghrelin on Osteoblasts

**Figure 4:**
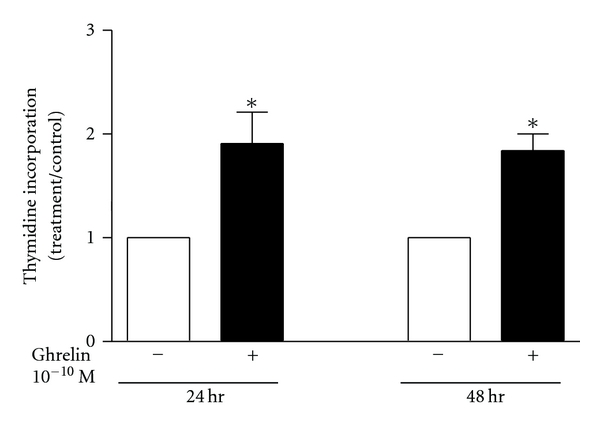
Ghrelin is mitogenic to primary human osteoblasts. Osteoblasts cultured from human trabecular bone were growth-arrested in 0.1% BSA for 24 hours. Ghrelin was added for 24 or 48 hours, and cells were pulsed with ^3^H-thymidine for the last 24 hours of the incubation. Data are mean ± SEM; *indicates *P* < 0.05 compared to untreated control by *t*-test.

**Figure 5:**
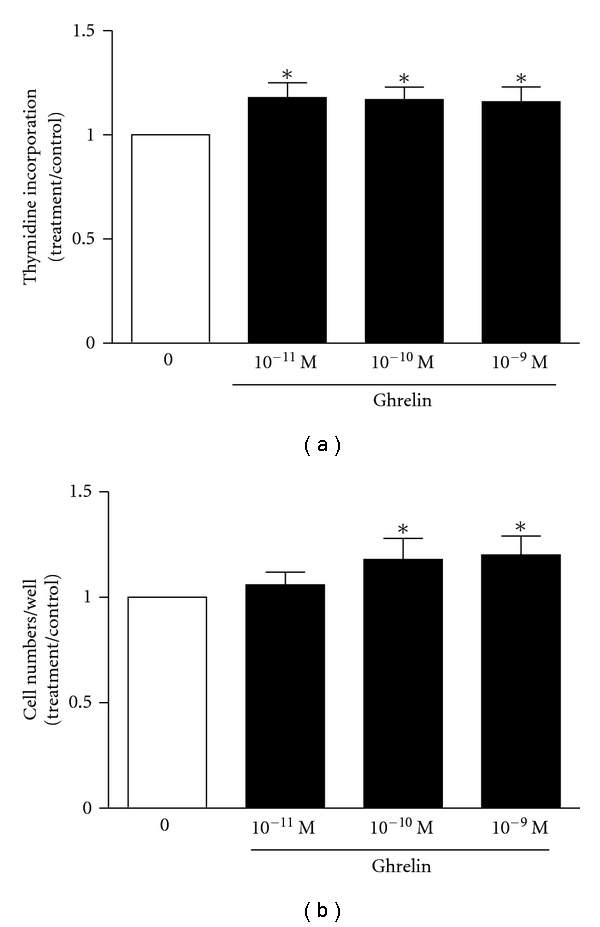
Ghrelin is weakly mitogenic to primary rat osteoblasts. Osteoblasts cultured from rat calvariae were growth-arrested in 0.1% BSA for 24 hours. Fresh media and experimental compounds were then added for a further 24 hours. (a) Thymidine incorporation: cells were pulsed with ^3^H-thymidine for the last 6 hours of incubation (ANOVA *P* = 0.018). (b) Cell counts (ANOVA *P* = 0.076). Data are mean ± SEM; *indicates *P* < 0.05 compared to untreated control by *t*-test.

Human primary osteoblasts incubated for 24 or 48 hours in the presence of 0.1 nM ghrelin showed a 2-fold increase in proliferation, as measured by thymidine incorporation (Figure 4). Ghrelin weakly induced cell proliferation in primary rat osteoblasts, with a 1.1-1.2-fold stimulation of thymidine incorporation (Figure 5(a)) and a similar, modest increase in cell numbers (Figure 5(b)). 

We also investigated ghrelin's effects on osteoblast differentiation. Primary rat osteoblasts were cultured for 21 days in media supplemented with L-ascorbic acid-2-phosphate and *β*-glycerophosphate, and mineralized bone nodule formation was measured by Von Kossa stain. Ghrelin had no effect on osteoblast differentiation in this assay (data not shown). The effect of ghrelin on osteoblast survival was investigated using a TUNEL assay. Apoptosis was induced in primary rat osteoblast by serum withdrawal, and the cells were incubated for a further 24 hours in the presence of ghrelin or controls. There was no significant change in the number of apoptotic bodies between the treatment and control wells, indicating that ghrelin had no effect on cell survival in this experimental system (data not shown). 

### 3.3. The Expression of Ghrelin Receptor, GHSR, in Bone Cells from Human, Rat, and Mouse Origin

Quantitative PCR was used to determine the expression of GHSR in bone cells. The GHSR TaqMan probe we used is designed to hybridize to a common sequence which is included in both of the alternatively splices products of the gene: GHSR1a and GHSR1b. We tested the expression of GHSR in RNA extracted from human primary osteoblasts and bone marrow samples and found that the expression varies among individuals, with 3 out of 10 osteoblast samples and 4 out of 16 bone marrow samples showing a positive amplification signal. Rat primary osteoblast and mouse bone marrow cells showed no expression of *Ghsr*. We also tested the mouse osteoblastic cell line MC3T3-E1, collecting RNA samples on different days during the differentiation of the cells into mature osteoblasts in vitro and found no expression of *Ghsr*. RNA samples collected from the murine macrophage cell line RAW_264.7_, which differentiate in vitro into osteoclasts, were also negative for *Ghsr*. RNA extracted from mouse hypothalamic tissue which was used as positive control showed high levels of *Ghsr *expression (Table 1).

## 4. Discussion

The gastric hormone ghrelin is primarily secreted from the stomach in response to hunger or fasting and it induces growth hormone secretion and food intake, most likely via activation of type 1a receptors in the arcuate nucleus of the hypothalamus. Here we investigated the direct impact of ghrelin on bone cells to expand on previous in vitro studies of ghrelin and show the first data regarding ghrelin's impact on osteoclasts in vitro. The finding that ghrelin stimulates mature osteoclast activity to resorb bone suggests a possible direct link between increased levels of circulating ghrelin and stimulation of bone resorption induced by fasting.

Ghrelin does not appear to be a primary requirement for bone formation and remodeling. In mouse models lacking either ghrelin or the ghrelin receptor, there are no known changes to the skeleton. Mice lacking ghrelin [36] are normal in size and weight compared to their littermate controls, showing neither dwarfism nor obesity and no change in bone mineral content or density. Mice lacking the ghrelin receptor have normal body size with slightly reduced body weight though there is no change in their bone mineral content or density [37]. The lack of changes in these null models suggests a redundancy in the ghrelin-growth hormone system. Overexpression of ghrelin from the brain and stomach of transgenic mice does not induce any changes in body size, though these animals have increased food intake, while the impact on bone is unknown [38]. 

Our study shows stimulation of osteoblast proliferation in primary cells derived from rat calvariae and from human bone. Stimulation of proliferation in similar cultures from rat and human origin has been demonstrated before [24, 27, 28]. However, the finding that ghrelin had no effect on osteoblast differentiation and apoptosis varies from what has been shown by some of the other groups: increased osteoblast differentiation was determined in rat primary osteoblasts and in MC3T3-E1 cells [23, 24, 28], whereas in the human osteoblast cell line SV-HFO ghrelin had no effect on differentiation [27], similar to what we found in the current study. Kim et al. [23] showed that ghrelin inhibits apoptosis in MC3T3-E1 cells, in contrast to our observations that ghrelin had no effect on apoptosis in primary rat osteoblasts. A study of the effect of ghrelin on apoptosis in rat osteoblasts and various other primary cells of rat and human, as well as in human aldosteronoma and human adrenocortical carcinoma-derived cell lines, showed that ghrelin did not affect apoptotic rate of normal cells, but significantly enhanced apoptosis in tumor-derived cells [26]. Thus, the effect of ghrelin on apoptosis appears to depend on the cells tested, with reports of inhibition, activation, and no effect on apoptosis in different cells. 

Ghrelin receptors are widely distributed and have previously been shown to be present in bone cells and tissue by reverse-transcription PCR, western blots and immunohistochemistry [23–25, 28]. We used real-time PCR to test the expression of *Ghsr* in primary rat osteoblasts and mouse bone marrow cells, and in the mouse cell lines MC3T3-E1 and RAW_264.7_, but failed to find any expression of the ghrelin receptor. This was not due to technical problems, as expression was robust in hypothalamic positive controls. In contrast to the lack of expression in bone cells of rodents, we found that bone cells from humans expressed the ghrelin receptor, although we could only detect low levels of expression in less than half of the osteoblast and bone marrow samples tested. The expression of GHSR in cells from 26 different patients showed individual variability that could perhaps explain some inconsistencies in results from different studies. Delhanty et al. [27] used TaqMan assays that were designed to distinguish between GHSR1a and GHSR1b to determine the expression of GHSR in bone samples from two patients and found that only the GHSR1b transcript was detectable. It would be of interest to test if the human RNA samples used in our study also express exclusively the GHSR1b splice variant. 

The fact that ghrelin promotes proliferation of rat osteoblasts though these cells do not express the ghrelin receptor may suggest the existence of an alternative signaling pathway for ghrelin and raises the possibility of unreported negative results or age-dependent expression, as seen in adipocytes [39]. Previous studies have also indicated that an additional, GHSR1a-independent pathway might be activated by ghrelin in a cardiomyocyte cell line not expressing GHSR [40]. Here, ghrelin was found to promote proliferation of human osteoblasts, although these cells express only the GHSR1b isoform, which is considered to be inactive [27]. In a unilateral bone marrow infusion experimental system, Thompson et al. [14] showed that des-acyl and n-octanoylated ghrelin were active whereas a potent synthetic GHSR1a agonist was ineffective, and so the authors suggest a pathway involving a receptor other than GHSR1a. At least in the case of des-acyl ghrelin, Chen et al. [41] found that it may act via a corticotropin releasing factor receptor, but there do not currently appear to be any studies investigating an alternative signaling pathway for n-octanoylated ghrelin. This is a worthwhile goal for the future of this field.

Since its discovery in 1999 [5], ghrelin has been the focus of a large number of studies. The activity of ghrelin in bone has been investigated in vivo and in vitro, but the results have not always been consistent. Our investigations of the effects of n-octanoylated-ghrelin in bone cells in vitro provide the first data in support of a direct effect of ghrelin to promote bone resorption by osteoclasts. 

## Figures and Tables

**Table 1:** Ghrelin receptor expression in bone.

This study	Method	Result
*Primary tissue*		
Human osteoblasts (*n* = 10)	Quantitative PCR	3/10 positive
Human bone marrow (*n* = 16)		4/16 positive
Mouse bone marrow		Negative
Rat osteoblastic cells		Negative
Mouse hypothalamus		Positive
*Cell lines*		
Mouse osteoblastic MC3T3-E1		Negative
Mouse macrophage RAW_264.7_		Negative

Kim et al. [23]		

*Primary Tissue*		
Rat osteoblastic cells	Western blot	Positive
Mouse osteoblastic cells		
Rat brain		
*Cell lines*		
Mouse osteoblastic MC3T3-E1		Positive
Mouse preadipocytic 3T3-L1	RT-PCR, Western blot	
Rat ROS17/2.8 osteogenic sarcoma		
Rat UMR-106 osteogenic sarcoma		

Maccarinelli et al. [24]		

*Primary Tissue*		
Rat osteoblastic cells	RT-PCR, Western blot	Positive

Ueberberg et al. [25]		

*Primary Tissue*		
Human bone marrow	Quantitative PCR, IHC	Positive

Delhanty et al. [27]		

*Primary Tissue*		
Human bone marrow (*n* = 2)	Quantitative PCR	Positive

Fukushima et al. [28]		

*Primary Tissue*		
Rat osteoblastic cells	RT-PCR, IHC	Positive
Rat femur	IHC	Positive
*Cell lines*		
Rat UMR-106 osteogenic sarcoma	RT-PCR	Positive

Guan et al. [35]		

*Primary Tissue*		
Rat bone marrow	RPA	Negative

RT-PCR: reverse transcription PCR. IHC: immunohistochemistry. RPA, ribonuclease protection assay.
